# Simultaneous Increases in Proliferation and Apoptosis of Vascular Smooth Muscle Cells Accelerate Diabetic Mouse Venous Atherosclerosis

**DOI:** 10.1371/journal.pone.0141375

**Published:** 2015-10-21

**Authors:** Suning Ping, Yuhuang Li, Shuying Liu, Zhengyu Zhang, Jingjing Wang, Yuhuan Zhou, Kefeng Liu, Jintao Huang, Dadi Chen, Junmei Wang, Chaohong Li

**Affiliations:** 1 Department of Histology and Embryology, Zhongshan School of Medicine, Sun Yat-sen University, Guangzhou, Guangdong Province, China; 2 Experimental Center, Zhongshan School of Medicine, Sun Yat-sen University, Guangzhou, Guangdong Province, China; 3 Department of Histology and Embryology, Basic Medical College, Guangzhou Medical University, Guangzhou, Guangdong Province, China; University of Sassari, ITALY

## Abstract

**Aims:**

This study was designed to demonstrate simultaneous increases in proliferation and apoptosis of vascular smooth muscle cells (VSMCs) leading to accelerated vein graft remodeling and to explore the underlying mechanisms.

**Methods:**

Vein grafts were performed in non-diabetic and diabetic mice. The cultured quiescent VSMCs were subjected to mechanical stretch stress (SS) and/or advanced glycosylation end products (AGEs). Harvested vein grafts and treated VSMCs were used to detect cell proliferation, apoptosis, mitogen-activated protein kinases (MAPKs) activation and SM-α-actin expression.

**Results:**

Significantly thicker vessel walls and greater increases in proliferation and apoptosis were observed in diabetic vein grafts than those in non-diabetic. Both SS and AGEs were found to induce different activation of three members of MAPKs and simultaneous increases in proliferation and apoptosis of VSMCs, and combined treatment with both had a synergistic effect. VSMCs with strong SM-α-actin expression represented more activated JNKs or p38MAPK, and cell apoptosis, while the cells with weak SM-α-actin expression demonstrated preferential activation of ERKs and cell proliferation. In contrast, inhibition of MAPKs signals triggered significant decreases in VSMC proliferation, and apoptosis. Treatment of the cells with RNA interference of receptor of AGEs (RAGE) also resulted in significant decreases in both proliferation and apoptosis.

**Conclusions:**

Increased pressure-induced SS triggers simultaneous increases in proliferation and apoptosis of VSMCs in the vein grafts leading to vein arterializations, which can be synergistically accelerated by high glucose-induced AGEs resulting in vein graft atherosclerosis. Either SS or AGEs and their combination induce simultaneous increases in proliferation and apoptosis of VSMCs via different activation of three members of MAPKs resulting from different VSMC subtypes classified by SM-α-actin expression levels.

## Introduction

Coronary artery bypass surgery involving vein grafts is the most common surgical revascularization strategy in patients with ischemic heart disease. However, the long-term efficacy remains limited because about 50% of venous grafts are closed 10 years after surgery [1, 2], especially in patients with diabetes[3]. Vein grafts are implanted into arterial pressures, where they are subjected to sudden increases in biomechanical forces in the form of stretch stress (SS). The stress may stimulate the wall of the grafted vessels and may activate intracellular signal pathways, leading to vascular cell differentiation, migration, proliferation and apoptosis [4]. This can cause neointimal hyperplasia or atherosclerosis [5], proceeding to atheroma in vein grafts and ultimately serious clinical problems. The pathogenic mechanisms of atheroma remain elusive and few effective techniques are available to prevent this event. Increasing data have demonstrated that rates of obstructive atherosclerosis in vein grafts are closely correlated to preoperative blood glucose levels (present in both type I and type II diabetes) and the development of lesions can be predicted by high advanced glycosylation end-products (AGEs) levels. Our previous study demonstrated that streptozocin (STZ)-induced hyperglycemia caused significant increases of AGEs in serum and vein grafts which led to rapid vein graft atherosclerosis [5]. AGEs are proteins induced by high blood glucose (diabetes) *via* non-enzymatic glycation and oxidation [6]. However, the veins of these mice themselves have no change in structure and function. This implies that increased pressure-induced SS initiates the vascular remodeling signals, which can be further amplified by AGEs leading to rapid vein graft atherosclerosis other than arterializations eventually. This also means that molecular mechanisms by which single or combined simulation of SS and AGEs triggers vascular remodeling are largely different. Unfortunately, the reports concerning combination of SS and AGEs are quite inadequate.

The rapid and reversible activation of mitogen-activated protein kinases (MAPKs) can be strongly stimulated by growth factors [7], cytokines [8] and stresses [9]. Three major members of MAPK family have been identified, including the extracellular signal-regulated kinases (ERKs), c-Jun NH2-terminal protein kinases (JNKs) or stress-activated protein kinases (SAPKs) and p38MAPKs [10]. The activation of ERKs is closely associated with cell proliferation [7, 11], and the activated JNKs and p38MAPK eventually lead to cell apoptosis [12, 13]. Mechanical stretched stress [14], ox-LDL [15] and AGEs [5] can trigger simultaneous activation of all three members of MAPK family, indicating simultaneous initiation of both proliferative and apoptotic signals. However, all these results derived from Western blot analysis, which provides results from all cells in the cultures. So, it is necessary to know the in situ activation profiles of three members of the MAPKs in the individual cells in cultures and vein grafts in response to the same stimuli, but no such relevant report is available so far.

Cell proliferation and apoptosis play equally important roles in vascular remodeling [16], cancer [17] and embryonic development [18]. In our previous studies, SS not only induced increased VSMC apoptosis *via* the p38MAPK pathway, but also caused VSMC proliferation *via* ERK signaling in the presence and absence of AGEs, oxLDL, and norepinephrine. This phenomenon (death and survival) seems to be contradictory, and the real appearance across the individual cells is still unknown. Why the cultured cells or cells in vein grafts represent different cell fates and which factors decide the cell’s fates in response to the same extracellular stimuli remain to be demonstrated.

Here, we hypothesize that cell proliferation and apoptosis exist simultaneously in cultured cells or vein grafts due to different activation of MAPKs, which are closely associated with subtypes of VSMCs. We establish a novel approach to simultaneously observe proliferative, apoptotic and resting cells in the same field of the cultures and the vein grafts. These results would widen our present knowledge for understanding molecular mechanisms of development and treatment of vascular remodeling and disease, especially in the diabetic setting.

## Materials and Methods

### Mouse models of vein graft

All experimental procedures were similar to the reported papers [19] with slight modification. Three-month-old male C57BL/6J mice were purchased from the animal facility center of Sun Yat-sen University, maintained on a light/dark (12/12 h) cycle at 25°C and received food and water ad libitum before experiments. The mice were divided into a non-diabetic group (ND) and a diabetic group (D) (N = 50, respectively). In the diabetic group, each mouse received seven consecutive daily injection of STZ (Sigma) (i.p. 50 mg/kg), while in non-diabetic group, mice were injected with citrate buffer as a control. Blood glucose levels of two groups were measured one week later. Levels above 288 mg/dl were considered as indicative of diabetes. Then the mice were subjected to vein graft surgery as previously reported. In brief, the vena cava of the isogenic donor mouse was grafted into dissected right common carotid artery of the recipient mouse. Vigorous pulsations confirmed successful engraftment. The mouse was anesthetized by sodium pentobarbital (i.p. 50 mg/kg). At same time atropine sulfate was also administrated at a dose of 1.7 mg/kg body weight to keep the respiratory tract clear by reducing salivary secretion. Allow a 10-min interval between the injection and operation to make sure the muscle tension was in a relaxed condition. The surgery would be finished within 40 minutes to relieve the pain. After the surgery, the recovery time was variable, from 30 min to 2 h. Warm blanket and oxygen inhalation were applied. Sufficient food and water were supplied to the mice. All animal procedures were consistent with the National Institute of Health Guide for the Care and Use of Laboratory Animals and approved by the Animal Care and Use Committee of Sun Yat-sen University. The vein grafts were harvested 8 weeks after operations and fixed with 4% paraformaldehyde. Paraffin-embedded samples were made into 4 μm-thick sections for further analysis.

### *In situ* immunofluorescent staining of vein grafts

Paraffin-embedded sections were subjected to immunofluorescent staining in accordance with the protocols provided by Abcam (www.abcam.com/technical) with slight modifications. Briefly, the sections were incubated with primary antibodies mixed in 0.3% Triton X-100 overnight at 4°C, followed by incubation with CY3- and FITC-conjugated secondary antibodies. The nuclei were counterstained with 4′, 6-diamidino-2-phenylindole (DAPI). For *in situ* detection of different activation profiles of three members of MAPKs in the vein grafts, primary antibody including p-ERKs, p-JNKs, and p-p38MAPK were performed. For simultaneous detection of proliferation and apoptosis, Ki67 antibody was performed, and then TUNEL kit was stained according to the instructions. For simultaneous detection of proliferative or apoptotic cells with SM-α-actin expression in vein grafts, the treated sections were incubated with SM-α-actin and Ki67 antibody or TUNEL kit at the same time. The slides were inspected and photographed using fluorescence microscopy (Olympus, Tokyo, Japan). Ki67-, TUNEL-positive cells and total nuclei were counted and analyzed by two independent researchers blinded to the specimen groups.

### Cell culture and treatment

VSMCs were isolated by enzymatic digestion of the aortas of C57BL/6J mice using a modified version of a previously described procedure [20]. The isolated cells grown in gelatin coated 6-well culture plates with silicone elastomer-bottom were maintained in a humidified atmosphere of 5% CO_2_ with growth medium (DMEM + 10% fetal calf serum + 100 μM streptomycin +100 U/ml penicillin). Cells achieving 80% confluence were serum-starved for 48 h and subjected to SS with Cyclic Stress Unit in the absence or presence of AGEs according to the procedures previously described [5, 21]. Cyclic Stress Unit, a modification of the unit initially described by Banes *et al*.[22], consisted of a controlled vacuum unit and a base plate to hold the culture plates (FX3000 AFC-CTL, Flexcell). A vacuum (15 to 20 kilopascals) was repeatedly applied to the elastomer-bottomed plates via the base plate, which was placed in a humidified incubator with 5% CO2 at 37°C. Cyclic deformation (60 cycles/min) and 10% elongation of elastomer-bottomed plates were used. This model of the apparatus generates a homogeneous stretch stress on the membrane. Preparation and identification of AGEs were similar to our previous report [23]. Pretreatment of the cells with inhibitors (PD98059 for MEK, SP600125 for JNK1/2, SB202190 for p38MAPK, PDTC for NF-κB, Z-DEVD-FMK for Caspase-3) (Cell Signal Tech.) were respectively utilized to determine the effects of these intracellular molecules on signal pathways and cell functions.

### RNA interference

The RAGE small interfering RNA (siRNA-RAGE) targeted duplex sequences [NM_007425] (Sense: 5′- GAGACACCCUGAGACGGGACUCUUU-3′; Antisense: 5′- AAAGAGUCCCGUCUCAGGGUGUCUC-3′); and non-targeting siRNA duplex sequences as a negative control were purchased from Life Technologies. VSMC transfection was performed according to the manufacturer’s recommendations. Serum-starved VSMCs were subjected to SS in the absence or presence of AGEs for 10 min and harvested for Western blot analysis. For the detection of cell proliferation and apoptosis, the cells were treated with SS and/or AGEs for 1 h, and then cultured for an additional 23 h.

### Western blotting

The cultured VSMCs pretreated with or without inhibitors were subjected to SS (10% elongation) and/or AGEs (100 μg/ml) and then harvested in lysis buffer with protease inhibitors. The lysate suspension was centrifuged and protein concentration was assessed using a Bio-Rad protein assay. Heat-denatured proteins (100 μg/sample) were resolved by SDS-PAGE and electrophoretically transferred to nitrocellulose membranes. These membranes were probed, stripped and then re-probed time and again with antibodies against p-ERKs, p-JNKs, p-p38MAPK, ERK, JNK, p38MAPK, p-NF-κB/p65(Ser536), NF-κB, Caspase-3 (Cell Signal Tech.), and β-actin (Santa Cruz) finally. The bands were visualized using the enhanced chemiluminescence (ECL) detection system. Total MAPKs were used for internal control to phosphorylated MAPKs, NF-κB was used for internal control to phosphorylated NF-κB, and β-actin was used for internal control to cleaved Caspase-3 and RAGE. Graphs were analyzed by ImageJ 1.32 program for the level of specific induction.

### *In situ* detection of proliferative, apoptotic, and resting VSMCs and phosphorylated MAPKs, and SM-alpha-actin expression in cultures

*In situ* proliferation and apoptosis of cultured VSMCs were examined by immunofluorescent staining with Ki67 antibody (Santa Cruz) and a TUNEL kit (Roche, Basel, Switzerland) in the presence of DAPI. Briefly, the treated cells (SS, 10% elongation and/or AGEs, 100 μg/ml, for 1 h, and continuously cultured for additional 23 h) were stained with Ki67 and corresponding CY3-conjugated secondary antibody (Jackson ImmunoResearch, U.S.), then with the TUNEL kit according to the kit’s instructions. The nuclei were counterstained with DAPI. The proliferative, apoptotic cells were identified by Ki67-positive (red color) and TUNEL-positive (green color) staining. Total nuclei (DAPI staining, blue color) and Ki67- or TUNEL-positive cells were counted and analyzed by two independent researchers blinded to the specimen groups. The proliferative and apoptotic index was calculated as the percentage of active proliferative or apoptotic cells versus the total cell count. Except for proliferative and apoptotic cells, all remaining cells (DAPI staining only) were considered as resting cells. For the detection of SM-α-actin expression and phosphorylation of three members of MAPKs, the cultured VSMCs were treated with SS and/or AGEs for 10 min, and then incubated with SM-α-actin and p-ERKs, or p-JNKs or p-p38MAPK antibodies at the same time and corresponding CY3 and FITC-conjugated secondary antibody. Nuclei were also counterstained with DAPI. The cells were inspected and photographed using fluorescence microscopy (Olympus, Tokyo, Japan).

### *In situ* detection of NF-κB translocation in cultured VSMCs

*In situ* NF-κB translocation of cultured VSMCs were examined by immunofluorescence with NF-κB antibody (Cell Signal Tech.) in the presence of DAPI. Briefly, the treated cells (SS, 10% elongation and/or AGEs, 100 μg/ml, for 30 min) were stained with NF-κB and corresponding CY3-conjugated secondary antibody (Jackson ImmunoResearch, U.S.). Pretreatment of the cells with inhibitors (PD98059 for MEK, SP600125 for JNK1/2, SB202190 for p38MAPK, PDTC for NF-κB, Z-DEVD-FMK for Caspase-3) (Cell Signal Tech.) were respectively utilized to determine the effects of these signal pathways on activation and translocation of NF-κB. The cells were inspected and photographed using fluorescence microscopy (Olympus, Tokyo, Japan).

### Statistical analysis

All analyses were performed with SPSS 16.0 (SPSS). Continuous variables were given as mean ± SEM and categorical variables were given as actual numbers and percentages. ANOVA was used for continuous variables and chi-square and Fisher exact tests were used for categorical variables. P <0.05 were considered statistically significant.

## Results

### Simultaneous increases in proliferation and apoptosis of vascular cells promote vein graft remodeling

**Fig 1:**
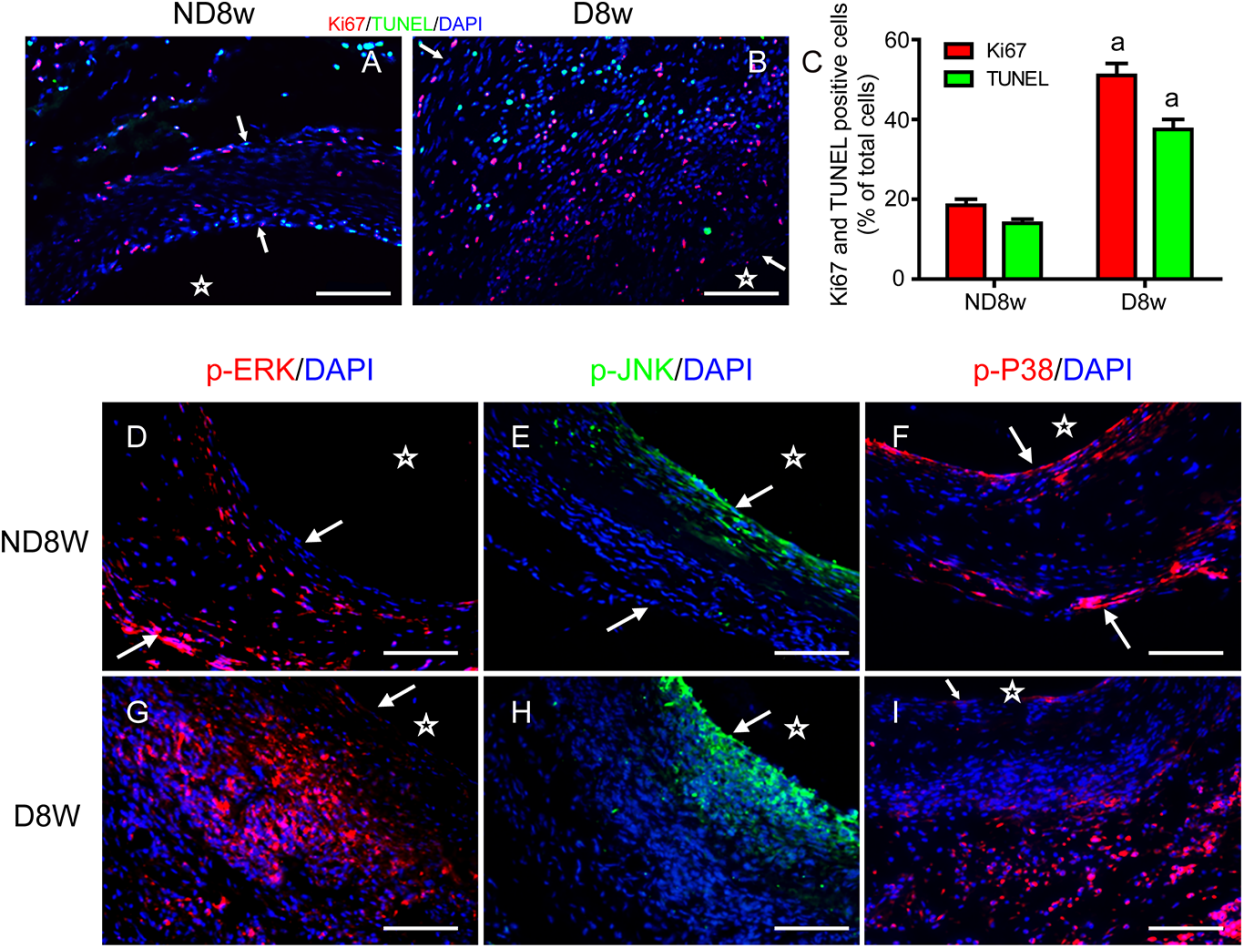
Simultaneous increases in proliferation and apoptosis of vascular cells in vein graft are associated with selective activation of MAPKs. **(A, B)** Immunofluorescence staining with Ki67 (red) and TUNEL (green) of both non-diabetic (ND) and diabetic (D) mice shows the simultaneous proliferation and apoptosis. **(C)** Graph bars show Ki67 and TUNEL positive ratios. **(D-I)** Immunofluorescence shows activation of ERKs, JNKs and p38MAPK in non-diabetic and diabetic mouse. Scale bars, 50 μm. Arrows and stars indicate the wall thickness and lumens of the vein grafts. All the experiments were independently repeated three times and presented as mean±SEM. a above bars represented the p<0.05 compared to the non-diabetic group, n = 3 (a for p value<0.05).

Our previous findings demonstrated that the wall thickness, AGEs deposition and Ki67 expression increased significantly in diabetic mouse vein grafts compared with those in non-diabetic. But there is currently no data for simultaneous proliferation and apoptosis in the same view of vein graft or other tissues. Here, using triple-labeled immunofluorescent staining, we showed that the proliferative (Ki67 positive, red), apoptotic (TUNEL positive, green) and resting (DAPI, blue only) cells were visible in vein grafts of both groups (Fig 1A and 1B). And the simultaneous proliferation and apoptosis were much higher in diabetic than those in non-diabetic mice (Fig 1C). Most of the proliferative and apoptotic cells were VSMCs, and distributed in all three layers of the vein grafts (S1 Fig). These results provide the first evidence that simultaneous increases in proliferation and apoptosis of VSMCs induced by rapid increases in blood pressure (mechanical stretch stress) could trigger the vein graft remodeling, while diabetes-related AGEs could further amplify the effects.

### Activation of ERKs, JNKs and p38MAPK differs in the vein grafts

To explore the mechanisms regulating the simultaneous proliferation and apoptosis, we measured the activation of MAPKs in the cells of vein grafts. As shown in Fig 1, ERK-activated cells (red) were visible in all three layers of diabetic and non-diabetic vein grafts. But the strong ERK-positive ones were mainly located in the inner side of the vein grafts (Fig 1D and 1G). Activated profiles of p38MAPK were mainly located in the two sides, especially in adventitia (Fig 1F and 1I). However, JNK-activated cells were predominantly located in neo-intima of the vein grafts (Fig 1E and 1H). These results suggest that simultaneous increases of proliferation and apoptosis in vein grafts cause vascular remodeling *via* selective activation of MAPK three members, which can be accelerated by diabetes-related AGEs.

### SS and/or AGEs induce simultaneous increases in proliferation and apoptosis in cultured VSMCs

**Fig 2:**
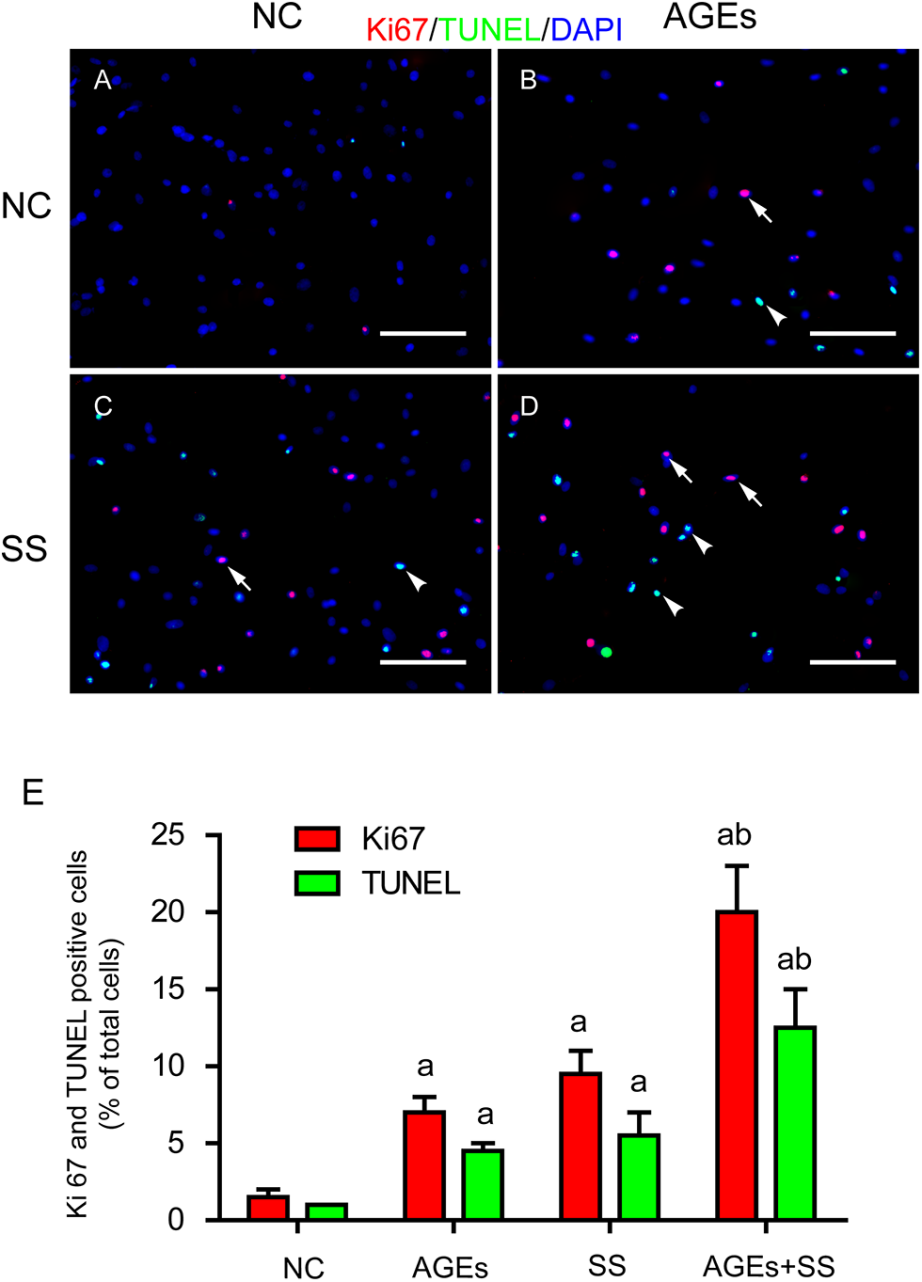
SS and AGEs synergistically promote simultaneous increases in proliferation and apoptosis of VSMCs. **(A-D)** Immunofluorescence analysis showed Ki67 and TUNEL positive cells of VMSCs in response to AGEs and SS. Either SS or AGEs could induce simultaneous increases in proliferation (Ki-67 positive, red, arrows) and apoptosis (TUNEL positive, green, arrowheads) compared to negative controls (NC), and combined stimulation with both had a synergistic effect. Scale bars, 50 μm. **(E)** Graph bars showed Ki67 and TUNEL positive ratios. All the experiments were independently repeated three times and shown as mean±SEM. a above bars represented the p<0.05 compared to NC group, and b represented the p<0.05 compared to AGEs or SS group, n = 3 (a and b for p value<0.05).

As mentioned above, we demonstrated the simultaneous proliferation and apoptosis of vein graft in vivo. Here, we further analyzed the changes of cultured VSMCs in vitro. Either SS (Fig 2C) or AGEs (Fig 2B) could induce simultaneous proliferation (Ki67 positive, red, arrows indicated) and apoptosis (TUNEL positive, green, arrowheads indicated) of VSMCs, and their combined stimulation had a synergistic effect (Fig 2D). Besides, there were also many resting cells (blue only). These results for the first time suggest that three fates of the cells (proliferative, apoptotic and resting cells) can be observed simultaneously in the same view of cultured VSMCs induced by SS and/or AGEs.

### SS and/or AGEs induce simultaneous activation of MAPKs in VSMCs

**Fig 3:**
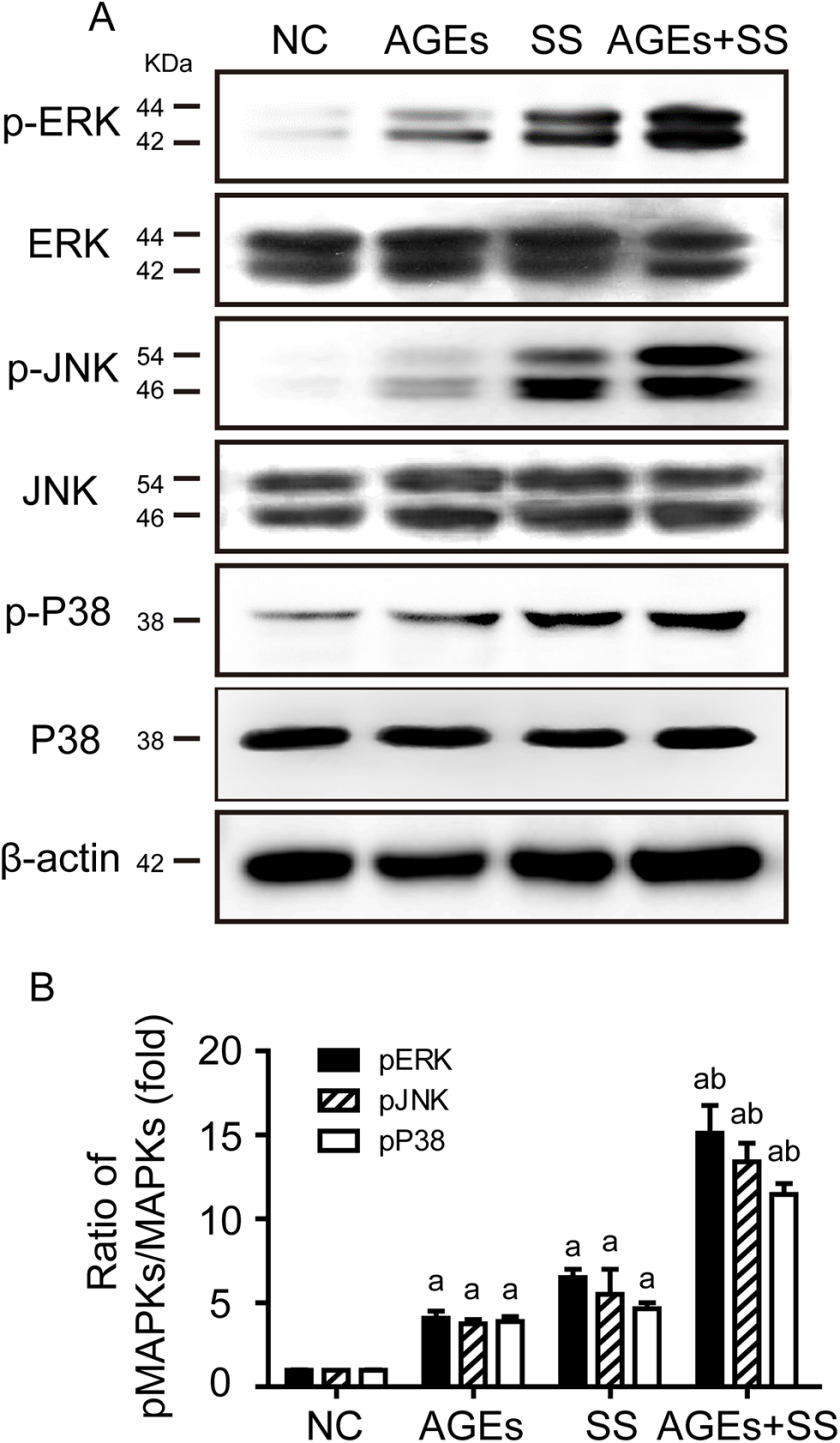
Combined SS and AGEs induce synergistic activation of three members of the MAPK subfamily in VSMCs. **(A)** Western blot analysis showed phosphorylation and total levels of ERK1/2, JNK1/2, and p38MAPK in VSMCs in response to AGEs and SS. **(B)** Graph bars showed densitometry analysis of phosphorylated ERK1/2, JNK1/2, and p38MAPK normalized with total MAPKs. All the experiments were independently repeated three times and shown as mean±SEM. a above bars represented the p<0.05 compared to NC group, and b represented the p<0.05 compared to AGEs or SS group, n = 3 (a and b for p value<0.05).

We previously reported that both SS and AGEs could induce ERK activation. It is unclear whether JNKs and p38MAPK can be activated by SS and/or AGEs. Using Western blot analysis, in the present study, we found that either SS or AGEs could induce simultaneous increases of phosphorylated ERKs, JNKs and p38MAPK in VSMCs, and the combined treatment with both had synergistic effects (Fig 3). Total expression of MAPKs was used as internal control in the experiment. These results provide the first evidence that SS and/or AGEs can simultaneously activate all three members of the MAPK subfamily.

### Selective activation of ERKs, JNKs and P38MAPK in the individual VSMCs in response to the same stimulation of SS and/or AGEs is closely associated with subtypes of VSMCs in cultures

**Fig 4:**
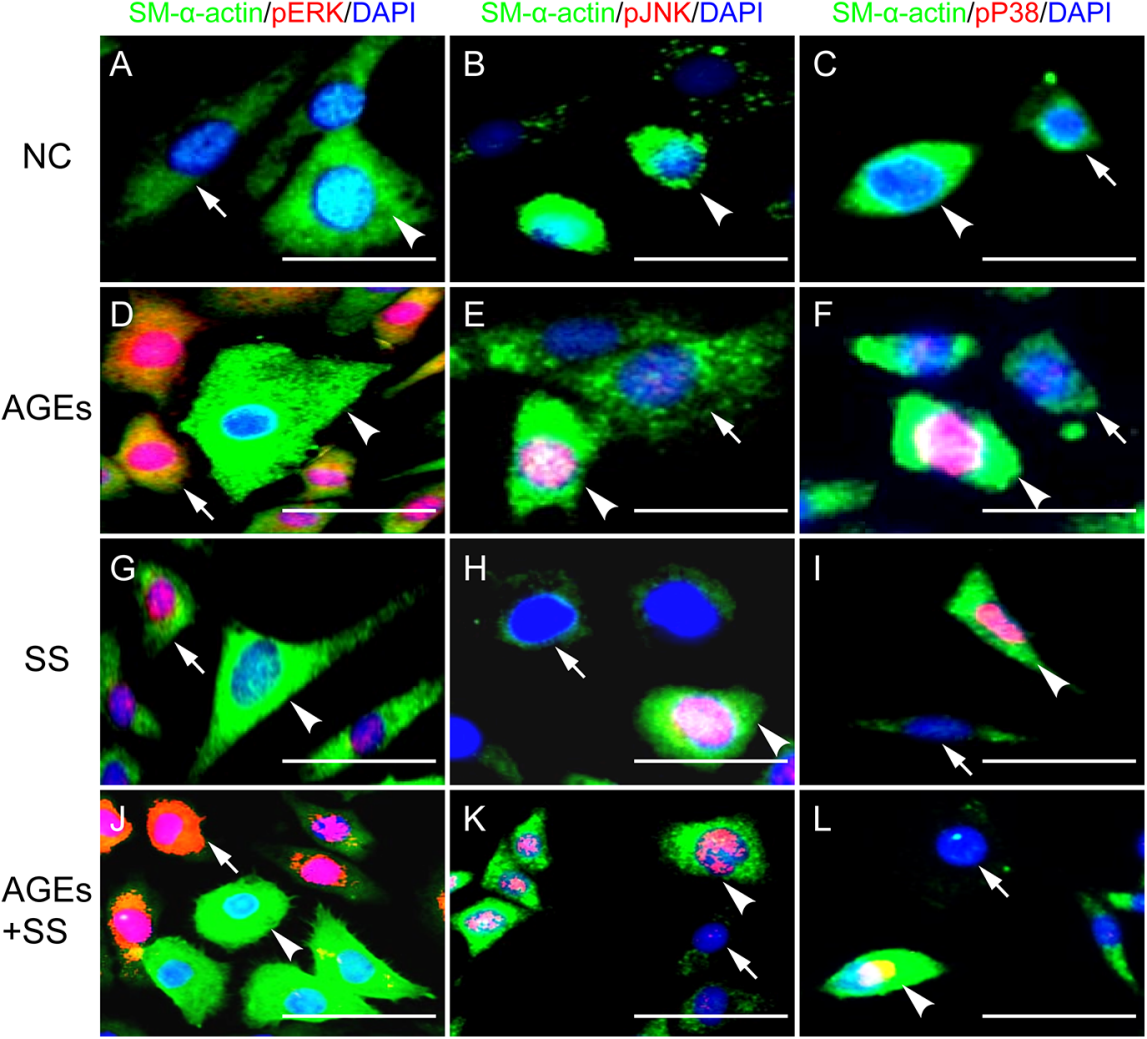
SS and AGEs induce selective activation of three members of the MAPK subfamily in individual VSMCs. Immunofluorescence was used to show selective activation of ERKs, JNKs and p38MAPK in single cells induced by SS and AGEs. VSMCs were marked with SM-α-actin, and the different expression levels (arrows indicated SM-α-actin weak positive cells and arrowheads indicated SM-α-actin strong positive cells) suggested subtypes existed. **(A, D, E, J)** Immunofluorescence showed ERK were preferentially activated in SM-α-actin weak positive cells. **(B, C, E, F, H, I, K, L)** JNK and p38MAPK were preferentially activated in SM-α-actin strong positive cells. Scale bars, 20 μm.

Results show that all the cells were SM-α-actin positive (green) (Fig 4), but the SM-α-actin expression levels were different, suggesting the subtypes existed in the VSMCs. In addition, SS and/or AGEs could significantly induce increased activation of MAPKs, but the activated profiles were different. In the cells with SM-α-actin strong expression (arrowheads), JNKs (Fig 4E, 4H and 4K) and p38MAPK (Fig 4F, 4I and 4L) were preferentially activated, and in the cells with SM-α-actin weak expression (arrows), more ERKs (Fig 4D, 4G and 4J) were activated. This heterogeneity of activation profiles was similar in all groups. These results suggest that MAPKs are differentially activated in response to the same stimuli, which are closely associated with the expression levels of SM-α-actin. The selective activation also might be related to the abovementioned simultaneous proliferative and apoptotic cells.

### Selective activation of MAPKs in the individual VSMCs leads to simultaneous increases in proliferation and apoptosis in response to SS and/or AGEs

**Fig 5:**
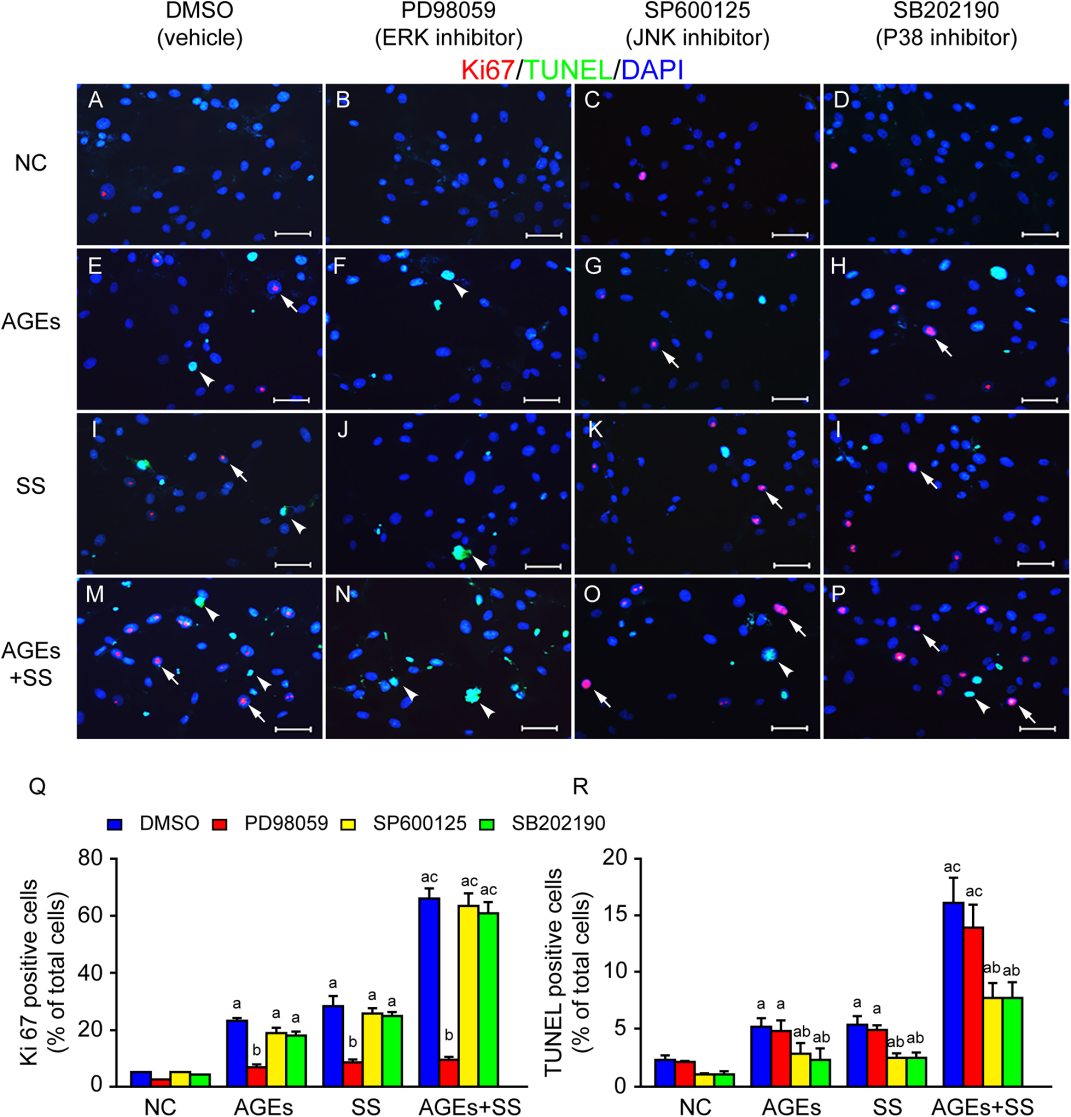
SS- and AGEs-induced simultaneous increases in proliferation and apoptosis of VSMCs are closely associated with selective activation of three members the MAPK subfamily. The cultured quiescent VSMCs were pretreated with DMSO, ERK inhibitor PD98059 (50 μM), JNK inhibitor SP600125 (20 μM), and p38MAPK inhibitor SB202190 (20 μM) for 1 h and then treated with SS and/or AGEs for 1 h and continually cultured for 23 h. **(A, E, I, M)** Either SS or AGEs could induce simultaneous increases in proliferation (Ki-67 positive, red, arrows) and apoptosis (TUNEL positive, green, arrowheads), and combined stimulation with both had a synergistic effect. **(B, F, J, N)** PD98059 completely suppressed cell proliferation while it had no effect on apoptosis. **(C, G, K, O, D, H, L, P)** SP600125 and SB202190 were found to significantly decrease apoptosis without affecting the cell proliferation. Scale bars, 20 μm. **(Q, R)** Graph bars showed Ki67 and TUNEL positive ratios. All the experiments were independently repeated three times and shown as mean±SEM. a above bars represented the p<0.05 compared to NC group, b represented the p<0.05 compared to DMSO group and c represented the p<0.05 compared to AGEs and SS group, n = 3 (a, b and c for p value<0.05).

To determine whether simultaneous proliferation and apoptosis of VSMCs were directly associated with selective activation of MAPKs, the cultured quiescent VSMCs were pretreated with inhibitors of ERKs (PD98059), JNKs (SP600125) and p38MAPK (SB202190), respectively, then stimulated with SS and/or AGEs. As expected, inhibition of ERKs completely suppressed cell proliferation while it had no effect on apoptosis (Fig 5B, 5F, 5J and 5N), and inhibition of JNKs and p38MAPK were found to significantly decrease apoptosis without affecting the cell proliferation (Fig 5C, 5G, 5K, 5O, 5D, 5H, 5L and 5P). These data suggest that selective activation of three members of MAPKs in the individual VSMCs in responses to stimulation by SS and/or AGEs leads to simultaneous increases in proliferation and apoptosis of VSMCs in the same field of cultures.

### JNKs/p38MAPK/Caspase-3 signaling plays an important role in mediating the apoptosis but not proliferation of VSMCs

**Fig 6:**
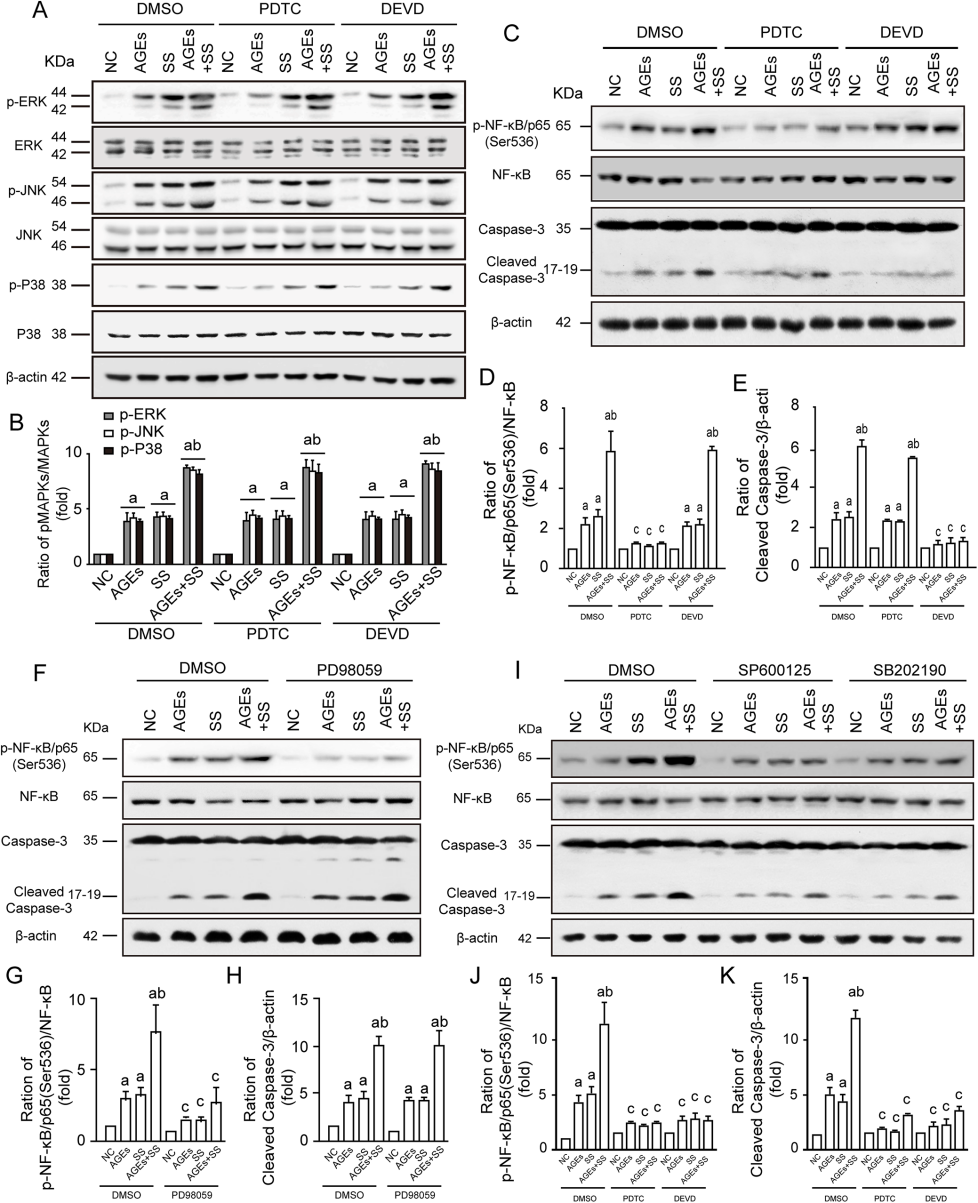
Relationship among MAPKs, NF-κB, and Caspase-3 in VSMCs induced by SS and AGEs. The cultured quiescent VSMCs were pretreated with DMSO, NF-κB inhibitor PDTC (20 μM) and Caspase-3 inhibitor Z-DEVD-FMK (20 μM), ERK inhibitor PD98059 (50 μM), JNK inhibitor SP600125 (20 μM) and p38 inhibitor SB202190 (20 μM) for 1 h and then treated with AGEs or SS as indicated above. **(A)** Western Blot analysis showed cells pretreated with PDTC and Z-DEVD-FMK had no effect on phosphorylation of MAPKs. **(B)** Graph bars showed densitometry analysis of MAPKs activation levels normalized with total MAPKs. **(C)** Western Blot analysis showed cells pretreated with PDTC suppressed the activation of both NF-κB/p65 (Ser536), and Z-DEVD-FMK also inhibited the Caspase-3 activation while had no effect on NF-κB/p65 (Ser536) phosphorylation of VSMCs. **(D)** Graph bars showed densitometry analysis of NF-κB/p65 (Ser536) activation levels after PDTC and Z-DEVD-FMK pretreatment normalized with NF-κB. **(E)** Graph bars showed densitometry analysis of Caspase-3 activation levels after PDTC and Z-DEVD-FMK pretreatment normalized with β-actin. **(F)** Western Blot analysis showed PD98059 significantly inhibited phosphorylation of NF-κB/p65 (Ser536), while it had no influence on Caspase-3 activation. **(G)** Graph bars showed densitometry analysis of NF-κB/p65 (Ser536) activation levels after PD98059 pretreatment normalized with NF-κB. **(H)** Graph bars showed densitometry analysis of Caspase-3 activation levels after PD98059 pretreatment normalized with β-actin. **(I)** Western Blot analysis showed NF-κB/p65 (Ser536) and Caspase-3 activation could be suppressed by both SP600125 and SB202190. **(J)** Graph bars showed densitometry analysis of NF-κB/p65 (Ser536) activation levels after SP600125 and SB202190 pretreatment normalized with NF-κB. **(K)** Graph bars showed densitometry analysis of Caspase-3 activation levels after SP600125 and SB202190 pretreatment normalized with β-actin. All the experiments were independently repeated three times and shown as mean±SEM. a above bars represented the p<0.05 compared to NC group, b represented the p<0.05 compared to AGEs and SS group and c represented the p<0.05 compared to DMSO group, n = 3 (a, b and c for p value<0.05).

Here, various inhibitors were used to determine the signal pathways between apoptosis and proliferation and activation of MAPKs. The results demonstrated that SS and/or AGEs could significantly increase activation of MAPKs, NF-κB/p65 and Caspase-3 (Fig 6A and 6C). NF-κB inhibitor (PDTC) had no effect on MAPKs (Fig 6A) and Caspase-3 inhibitor (DEVD) had no effect on activation of MAPKs and NF-κB/p65 (Fig 6A and 6C). In addition, inhibition of ERKs (PD98059) significantly suppressed NF-κB/p65 activation, but had no effect on Caspase-3 activation (Fig 6F). Inhibition of JNKs (SP600125) and p38MAPK (SB202190) significantly suppressed activation of both NF-κB/p65 and Caspase-3 (Fig 6I). We also used immunofluorescence detecting NF-κB translocation to confirm the results (S2 Fig). SS and/or AGEs could significantly induce the translocation of NF-κB from cytoplasm to nucleus, obviously aggregated in the periphery of the nucleus, or lied on the nucleus of the VSMCs. Inhibition of ERKs (PD98059), JNKs (SP600125), p38MAPK (SB202190) and NF-κB (PDTC) could obviously suppress the NF-κB translocation, while the inhibitor of Caspase-3 (DEVD) had no effect on NF-κB translocation induced by AGEs and/or SS. Consistent with these results, inhibition of NF-κB could significantly decrease both cell proliferation and apoptosis, while inhibition of Caspase-3 completely inhibited cell apoptosis without affecting cell proliferation (S3 Fig). The results suggest that Caspase-3 and NF-κB/p65 are important downstream molecules of JNKs and p38MAPKs, which lead to cell apoptosis, while ERKs are closely associated with VSMC proliferation.

### RAGE-mediated selective activation of MAPKs causes simultaneous proliferation and apoptosis of VSMCs

**Fig 7:**
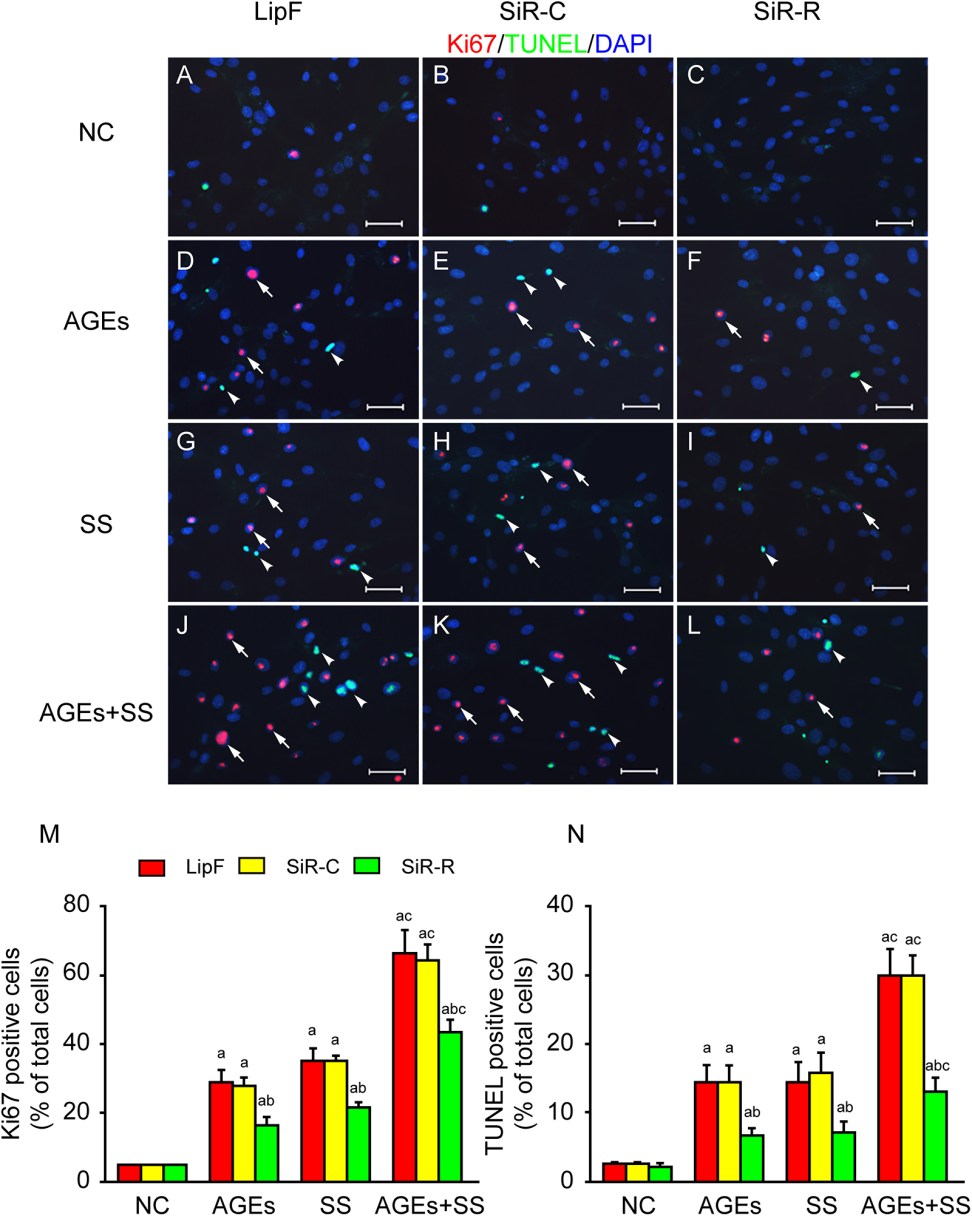
RAGE mediates simultaneous increases of proliferation and apoptosis of VSMCs. The cultured VSMCs were transfected with siRNA-RAGE (SiR), siRNA-control (SiC) and Lipofectamine 2000 (LIP) for 24 h and then serum-starved for additional 48 h. Cells were treated with AGEs and/or SS for 1 h and cultured for additional 23 h. **(A, D, G, J)** Immunofluorescence showed either AGEs or SS could increase the cell proliferation (red, arrows) and apoptosis (green, arrowheads), and the combination had a synergistic effect. **(B, E, H, K)** Immunofluorescence showed SiC had no effect on AGEs and SS induced increases of cell proliferation and apoptosis. **(C, F, I, L)** Immunofluorescence showed SiR significantly suppressed both proliferation and apoptosis of VSMCs in response to AGEs and SS. Scale bars, 20 μm. **(M, N)** Graph bars showed Ki67 and TUNEL positive ratios. All the experiments were independently repeated three times and shown as mean±SEM. a above bars represented the p<0.05 compared to NC group, b represented the p<0.05 compared to DMSO group and c represented the p<0.05 compared to AGEs and SS group, n = 3 (a, b and c for p value<0.05).

To establish the relationship between SS- and AGE-activated RAGE/MAPK signaling and proliferation and apoptosis, VSMCs were pretreated with RAGE-siRNA. RAGE-siRNA could significantly suppress RAGE expression of VSMCs (S4 Fig). As expected, RAGE-siRNA could trigger significant suppression of MAPKs activation compared with experimental controls (SiR-C) (S4 Fig). Likewise, SiRNA-RAGE could inhibit simultaneous increases in proliferation and apoptosis induced by SS and AGEs (Fig 7). The results suggest that RAGE can mediate SS- and AGE-initiated signals, resulting in selective activation of MAPKs and simultaneous proliferation and apoptosis of VSMCs.

## Discussion

In the present study, we proposed the idea that simultaneous increases in proliferation and apoptosis of VSMCs in the vein grafts induced by SS and/or AGEs trigger vein graft arterializations in non-diabetic mice and vein graft atherosclerosis in diabetic mice and established a new approach to confirm this hypothesis. Our data indicate that (1) simultaneous increases in proliferation and apoptosis of VSMCs are required for vein graft arterializations in non-diabetic mice and atherosclerosis in diabetic mice; (2) either SS or AGEs induces simultaneous increases in proliferation and apoptosis of cultured quiescent VSMCs, combination of both has a synergistic effect; (3) both SS and AGEs induce different activation of ERKs, JNKs and p38MAPK across the individual VSMCs leading to simultaneous increases in proliferation and apoptosis of cultured quiescent VSMCs; (4) different VSMC subtypes characterized by SM-alpha-actin expression in cultures and in the vein grafts respond differently to the same extracellular stimuli triggering simultaneous increases in proliferation and apoptosis via selective activation of ERKs, JNKs and p38MAPK; (5) Veins from the mice themselves have no change in structure, but the grafted veins change their structures soon after operation.

A balance between cell proliferation and apoptosis is important cellular events for normal development and tissue homeostasis[24]. Generally, the cell proliferation is thought to contribute significantly to the development of vascular remodeling [25]. Therefore, proliferation inhibition and apoptosis promotion become a preferential strategy for preventing and treating related diseases [26]. However, in this study, we conceptually proposed that simultaneous increases in proliferation and apoptosis of VSMCs trigger vein graft arterializations in non-diabetic mice and vein graft atherosclerosis in diabetic mice. We established the triple-labeled immunofluorescence approach to simultaneously exhibit coexistence of proliferative, apoptotic and quiescent cells in the same field of cultures in vitro and vein grafts in vivo. Both proliferation and apoptosis were absent in the vena cava from the mice themselves [5], but the grafted veins demonstrated simultaneous increases in proliferation and apoptosis of vascular cells (Fig 1). More proliferative and apoptotic cells could be found in the vein grafts of diabetic mice, which led to accelerated vein graft atherosclerosis. These results suggest that rapidly increased arterial pressure initiates cell proliferation and apoptosis within the mouse vein grafts, while high glucose-induced AGEs can synergistically amplify SS-initiated signaling. The in vivo results were confirmed by in vitro experiments. Either SS or AGEs could trigger the significant increase in simultaneous proliferation and apoptosis, and the combination had a synergistic effect. Thus, the effect of combination of SS and AGEs on vein graft remodeling should be very important. In support of our findings, Kalra and Miller[27] reported that cell proliferation and apoptosis occur simultaneously within the adventitia and media of the dog’s vein during the first week following grafting, but these data were obtained from normal dog, there were no significant pathological change in structure of the grafted veins. Although Salzberg et al [28] established a model of surgical vein grafting in a murine model of type 2 diabetes, they did not find significant cell proliferation (no Ki67-positive cells detected). It may result from different animal model. However, data from Lorusso *et al* [29] concerning human saphenous vein grafts are consistent with our reports. Thus, our results suggest that the clinical outcome of vein graft surgery depends on the simultaneously increased levels of proliferation and apoptosis of vascular cells. Simultaneous suppression of both proliferation and apoptosis should be considered as a new strategy for preventing and treating related diseases. In agreement with our findings, similar conclusions were proposed on tumor treatment studies [30].

We reported that the AGEs and SS could significantly increase ERK activation [5]. Consistent with previous results, the present study shows synergistic activation of three members in MAPK family (Fig 3). We further investigated the activation profiles across individual cells using triple-labeled immunofluorescence, since Western blotting results came from all cells cultured. For every single cell, MAPKs were significantly activated in all groups, but ERK was preferentially activated in some cells, while JNK and p38MAPK were activated in others (Fig 4). It is well known that ERK signaling is capable of modulating cell proliferation, and JNKs and p38MAPK are closely associated with apoptosis. Our results also confirmed the hypothesis. After pretreatment with ERK inhibitor (PD98059), cell proliferation was significantly inhibited but it had no effect on cell apoptosis. In contrast, JNK and p38MAPK inhibitor (SP600125 and SB202190) suppressed apoptosis without affecting cell proliferation (Fig 5). These results suggest that selective activation of MAPKs leads to the simultaneous proliferation and apoptosis of VSMCs in response to the same extracellular stimuli. However, the findings [31] of apoptosis-induced proliferation support our hypothesis that increased proliferation and apoptosis of cells co-existed in remodeling tissues. It has been identified as a process by which apoptotic cells can release mitogenic signals to promote surrounding cells proliferation in order to increase tissue repair and regeneration [32]. The more detailed mechanisms are unclear and whether it exists in our system remains to be elucidated.

NF-kappa B consists of a family of five transcription factors that form distinct protein complexes, which bind to consensus DNA sequences at promoter regions of responsive genes regulating cellular processes[33]. Increasing data have demonstrated that NF-κB activation plays key roles in cell migration, differentiation, inflammation, proliferation, and apoptosis [33–36]. In the present study, we found that NF-κB is a very important downstream molecule of MAPKs in VSMCs in response to stimulation of SS and/or AGEs. When stimulated with either SS or AGEs, increased NF-κB/p65 phosphorylation in the VSMCs was seen, and combination of both had a synergistic effect (Fig 6). Consistent with these results, SS and/of AGEs also could induce the translocation of total NF-κB from cytosol to nucleus. Compared with untreated cells, the total NF-κB obviously aggregated in the periphery of the nucleus, or lied on the nucleus of the VSMCs after stimulation, suggesting the obvious activation of NF-κB (S2 Fig). As expected, the activation of NF-κB induced by SS and/or AGEs could be significantly inhibited after the pretreatment with inhibitors of ERK (PD98059), JNK (SP600125), P38 (SB202190) and NF-κB (PDTC). However, the inhibitor of Caspase-3 (DEVD) had no effect in NF-κB activation. Interestingly, inhibition of NF-κB activation partially led to inhibition of increased proliferation and apoptosis of VSMCs induced by SS and/or AGEs (S3 Fig), while the inhibitor of Caspase-3 (DEVD) triggered complete inhibition of apoptosis. These results suggest that NF-κB-dependent and independent signal pathways mediate SS and/or AGEs-initiated signaling leading to simultaneous increases in proliferation and apoptosis.

The subtype of VSMCs plays a key role in the pathogenesis of vascular disorders [37]. In this study, our results showed that the cultured cells were all SM-α-actin positive, but the expression levels were different among individual cells, suggesting the existence of cell subtypes and heterogeneity. Besides, we also found that in the cells with strong SM-α-actin expression ERKs were preferentially activated, and more JNK and p38MAPK were activated in week SM-α-actin expression cells. These findings confirmed the conclusions of Qu et al. [38], and further indicated that the selective activation of MAPKs was closely associated with the heterogeneity of VSMCs. The cultured VSMCs isolated from the same mouse artery underwent several passages for cell expansion and were then used for experiments. Results showed the marked disorganization of SM-α-actin filaments in most of the cells, and only a small part remained normal, which was also in consistent with results observed by Chang *et al*.[39]. These data provide new evidence to support the conclusion that SM-α-actin might be an important drug target for the prevention and treatment of vascular remodeling and diseases, especially for diabetes.

**Fig 8:**
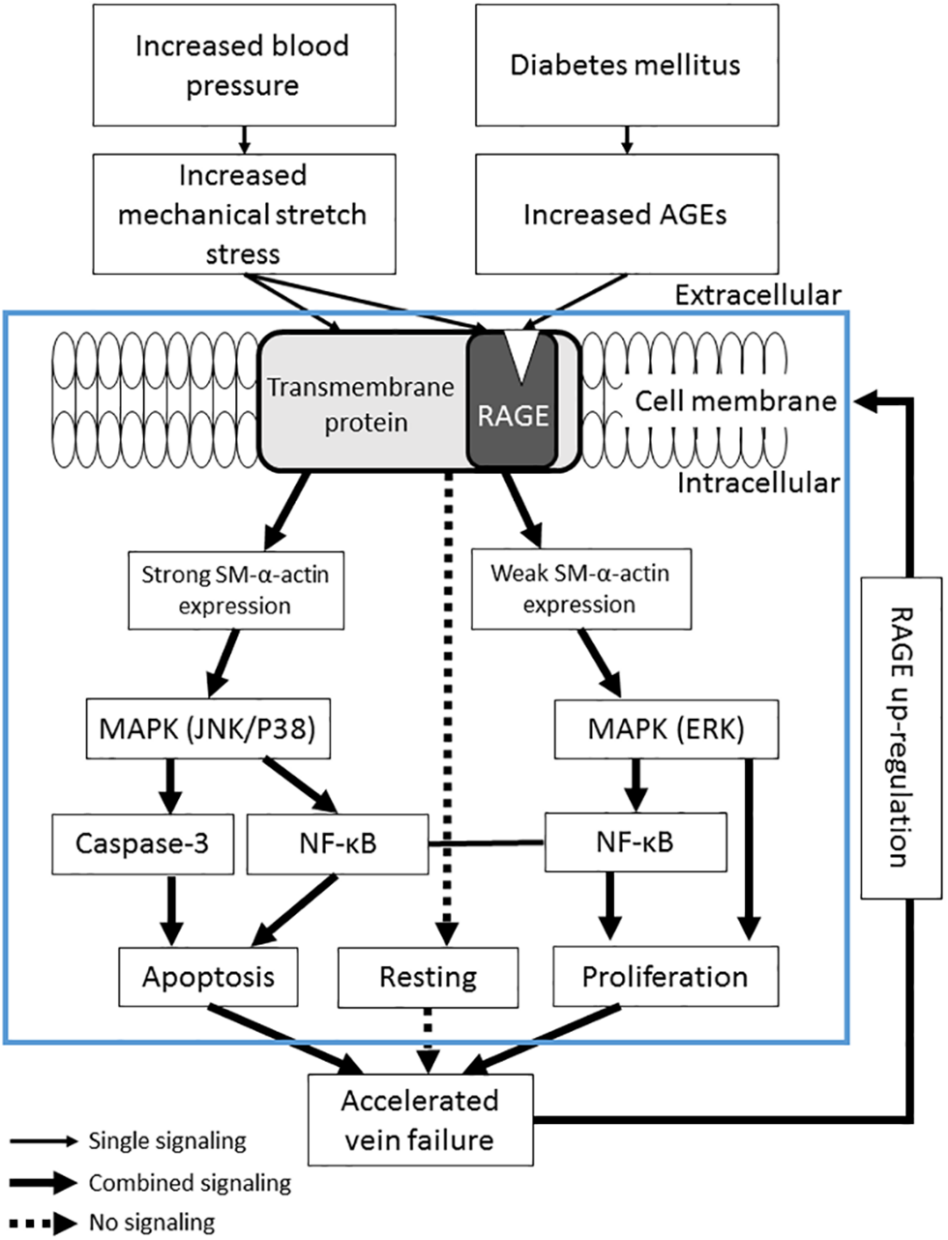
Potential signal pathway in the simultaneous increases of proliferation and apoptosis of VSMCs induced by SS and AGEs via RAGE/MAPK selective activation. Rapidly increased blood pressure triggers increased SS on the vein grafts. SS non-specifically activates RAGE and its downstream signal molecules, including selective activation of ERK/NF-κB and JNK/p38MAPK/NF-κB/Caspase-3 signaling, which leads to simultaneous increases in proliferation (Ki-67 expression) and apoptosis (TUNEL positive) of VSMCs. Hyperglycemia-induced numerous AGEs deposits on the vascular wall directly and specifically interacts with RAGE to further amplify the SS-initiated intracellular signaling molecules, which lead to synergistic increases in proliferation and apoptosis of VSMCs. Blocking RAGE and its downstream molecules might inhibit selective activation of MAPKs, leading to simultaneous decreases in the proliferation and apoptosis of VSMCs.

In conclusion, we report the simultaneous increases in proliferative, resting and apoptotic cells and their related MAPKs signal pathways in cultured VSMCs and vein grafts via triple-labeled immunofluorescence. Either SS or AGEs could significantly trigger the simultaneous increases in proliferation and apoptosis, and their combination had a synergistic effect. The different fates of VSMCs were caused mainly from selective activation of MAPKs, which was closely associated with VSMC subtypes with different levels of SM-α-actin expression (Fig 8). The in vitro results were confirmed by in vivo experiments. The veins were grafted into non-diabetic and diabetic carotid arteries for the same time period, the walls of the diabetic vein grafts became significantly thicker than those of non-diabetic vein grafts, and the cell proliferation and apoptosis were also more increased in the diabetic setting. In this way, this study reveals a novel mechanism that simultaneous increases in proliferation and apoptosis of VSMCs in vein grafts is a common characteristic of vascular remodeling and disease. These results would widen our present knowledge for understanding molecular mechanisms of development and treatment of vascular remodeling and disease, especially in the diabetic setting. They also could provide a new monitoring platform for organ remodeling and diseases involved in simultaneous cell apoptosis and proliferation, such as organ transplantation (e.g. vein graft), cancer therapy, fetus development, and so on.

## Supporting Information

S1 Fig Immunofluorescence shows increased proliferation and apoptosis of VSMCs in the vein grafts. **(A-D)** Immunofluorescence detecting cell proliferation and apoptosis located mainly in SM-α-actin positive cells in both non-diabetic and diabetic mouse. Scale bars, 20 μm.
(TIF)

S2 Fig NF-κB translocates to nucleus in cultured VSMCs induced by AGEs and/or SS. The cultured VSMCs were pretreated with DMSO, ERKs inhibitor PD98059, JNKs inhibitor SP600125, P38MAPK inhibitor SB202190, NF-κB inhibitor PDTC, and Caspase-3 inhibitor Z-DEVD-FMK for 1 h and then were treated with AGEs and/or for 30min. **(A, B, C, D)** Immunofluorescence showed either AGEs or SS could increase the NF-κB translocation (red, arrows), and the combination had a synergistic effect. **(E-T)** Immunofluorescence showed PD98059, SP600125, SB202190 and PDTC could significantly suppressed AGEs and SS induced increases of translocaiton. **(U, V, W, X)** Immunofluorescence showed Z-DEVD-FMK had no effect on AGEs and SS induced NF-κB translocation. Scale bars, 20 μm.
(TIF)

S3 Fig NF-κB and Caspase-3 involve in simultaneous increases in the proliferation and apoptosis of VSMCs induced by SS and AGEs. The cultured VSMCs were pretreated with DMSO, NF-κB inhibitor PDTC, and Caspase-3 inhibitor Z-DEVD-FMK for 1 h and then were treated with AGEs and/or for 1 h and continually cultured for 23 h. **(A, D, G, J)** Immunofluorescence showed either AGEs or SS could increase the cell proliferation (red, arrows) and apoptosis (green, arrowheads), and the combination had a synergistic effect. **(B, E, H, K)** Immunofluorescence showed PDTC significantly suppressed AGEs and SS induced increases of cell proliferation and apoptosis. **(C, F, I, L)** Immunofluorescence showed Z-DEVD-FMK significantly inhibited apoptosis of VSMCs while had no effect on cell proliferation. Scale bars, 20 μm. **(M, N)** Graph bars showed Ki67 and TUNEL positive ratios. All the experiments were independently repeated three times and shown as mean±SEM. a above bars are representing the p<0.05 compared to NC group, b represent the p<0.05 compared to DMSO group and c represent the p<0.05 compared to AGEs and SS group, n = 3 (a, b and c for p value<0.05).
(TIF)

S4 Fig Activation of MAPKs induced by AGEs, SS, or both is partially inhibited by siRNA-RAGE treatment. **(A)** Western blot analysis for RAGE detection of siRNA-RAGE transfected VSMCs. **(B)** Densitometry analysis of RAGE levels normalized with β-actin. **(C)** Western blot analysis for activation of MAPKs in siRNA-RAGE transfected VSMCs. **(D)** Densitometry analysis of MAPKs activation normalized with total MAPKs. All the experiments were independently repeated three times and shown as mean±SEM. a above bars are representing the p<0.05 compared to NC group, b represent the p<0.05 compared to siRNA-RAGE transfected group and c represent the p<0.05 compared to AGEs and SS group, n = 3 (a, b and c for p value<0.05).
(TIF)

## References

[1] Parang P, Arora R. Coronary vein graft disease: pathogenesis and prevention. The Canadian journal of cardiology. 2009;25(2):e57–62. Epub 2009/02/14. ; PubMed Central PMCID: PMCPmc2691920. 19214303 10.1016/s0828-282x(09)70486-6 PMC2691920

[2] FitzGibbon GM, Leach AJ, Kafka HP, Keon WJ. Coronary bypass graft fate: long-term angiographic study. Journal of the American College of Cardiology. 1991;17(5):1075–80. Epub 1991/04/01. . 2007706 10.1016/0735-1097(91)90834-v

[3] Harskamp RE, Lopes RD, Baisden CE, de Winter RJ, Alexander JH. Saphenous vein graft failure after coronary artery bypass surgery: pathophysiology, management, and future directions. Annals of surgery. 2013;257(5):824–33. Epub 2013/04/12. 10.1097/SLA.0b013e318288c38d . 23574989

[4] Li C, Xu Q. Mechanical stress-initiated signal transduction in vascular smooth muscle cells in vitro and in vivo. Cellular signalling. 2007;19(5):881–91. Epub 2007/02/10. 10.1016/j.cellsig.2007.01.004 . 17289345

[5] Li Y, Liu S, Zhang Z, Xu Q, Xie F, Wang J, et al RAGE mediates accelerated diabetic vein graft atherosclerosis induced by combined mechanical stress and AGEs via synergistic ERK activation. PLoS One. 2012;7(4):e35016 10.1371/journal.pone.0035016 22496883 PMC3322163

[6] Thornalley PJ. Dietary AGEs and ALEs and risk to human health by their interaction with the receptor for advanced glycation endproducts (RAGE)—an introduction. Mol Nutr Food Res. 2007;51(9):1107–10. Epub 2007/09/15. 10.1002/mnfr.200700017 . 17854008

[7] Liu G, Hitomi H, Hosomi N, Lei B, Nakano D, Deguchi K, et al Mechanical stretch augments insulin-induced vascular smooth muscle cell proliferation by insulin-like growth factor-1 receptor. Exp Cell Res. 2011;317(17):2420–8. Epub 2011/08/23. 10.1016/j.yexcr.2011.07.016 S0014-4827(11)00302-8 [pii]. . 21854769

[8] Cheng WP, Wang BW, Chen SC, Chang H, Shyu KG. Mechanical stretch induces the apoptosis regulator PUMA in vascular smooth muscle cells. Cardiovascular research. 2012;93(1):181–9. Epub 2011/10/25. 10.1093/cvr/cvr280 . 22021910

[9] Runchel C, Matsuzawa A, Ichijo H. Mitogen-activated protein kinases in mammalian oxidative stress responses. Antioxidants & redox signaling. 2011;15(1):205–18. Epub 2010/11/06. 10.1089/ars.2010.3733 . 21050144

[10] Rose BA, Force T, Wang Y. Mitogen-activated protein kinase signaling in the heart: angels versus demons in a heart-breaking tale. Physiological reviews. 2010;90(4):1507–46. Epub 2010/10/21. 10.1152/physrev.00054.2009 ; PubMed Central PMCID: PMCPmc3808831. 20959622 PMC3808831

[11] Weihs AM, Fuchs C, Teuschl AH, Hartinger J, Slezak P, Mittermayr R, et al Shock wave treatment enhances cell proliferation and improves wound healing by ATP release-coupled extracellular signal-regulated kinase (ERK) activation. The Journal of biological chemistry. 2014;289(39):27090–104. Epub 2014/08/15. 10.1074/jbc.M114.580936 ; PubMed Central PMCID: PMCPmc4175346. 25118288 PMC4175346

[12] Kim HJ, Kim JY, Lee SJ, Kim HJ, Oh CJ, Choi YK, et al alpha-Lipoic acid prevents neointimal hyperplasia via induction of p38 mitogen-activated protein kinase/Nur77-mediated apoptosis of vascular smooth muscle cells and accelerates postinjury reendothelialization. Arteriosclerosis, thrombosis, and vascular biology. 2010;30(11):2164–72. Epub 2010/09/11. 10.1161/atvbaha.110.212308 . 20829507

[13] Perez-Vizcaino F, Bishop-Bailley D, Lodi F, Duarte J, Cogolludo A, Moreno L, et al The flavonoid quercetin induces apoptosis and inhibits JNK activation in intimal vascular smooth muscle cells. Biochemical and biophysical research communications. 2006;346(3):919–25. Epub 2006/06/17. 10.1016/j.bbrc.2006.05.198 . 16777073

[14] Liu S, Li Y, Zhang Z, Xie F, Xu Q, Huang X, et al alpha1-Adrenergic receptors mediate combined signals initiated by mechanical stretch stress and norepinephrine leading to accelerated mouse vein graft atherosclerosis. Journal of vascular surgery. 2013;57(6):1645–56, 56.e1-3. Epub 2013/01/22. 10.1016/j.jvs.2012.09.061 . 23332241

[15] Zhang Z, Zhang M, Li Y, Liu S, Ping S, Wang J, et al Simvastatin inhibits the additive activation of ERK1/2 and proliferation of rat vascular smooth muscle cells induced by combined mechanical stress and oxLDL through LOX-1 pathway. Cellular signalling. 2013;25(1):332–40. Epub 2012/10/18. 10.1016/j.cellsig.2012.10.006 . 23072789

[16] Owens CD, Gasper WJ, Rahman AS, Conte MS. Vein graft failure. J Vasc Surg. 2015;61(1):203–16. Epub 2013/10/08. 10.1016/j.jvs.2013.08.019 S0741-5214(13)01531-0 [pii]. 24095042 PMC4391818

[17] Evan GI, Vousden KH. Proliferation, cell cycle and apoptosis in cancer. Nature. 2001;411(6835):342–8. Epub 2001/05/18. 10.1038/35077213 . 11357141

[18] Fisher SA, Langille BL, Srivastava D. Apoptosis during cardiovascular development. Circulation research. 2000;87(10):856–64. Epub 2000/11/14. . 11073880 10.1161/01.res.87.10.856

[19] Zou Y, Dietrich H, Hu Y, Metzler B, Wick G, Xu Q. Mouse model of venous bypass graft arteriosclerosis. Am J Pathol. 1998;153(4):1301–10. 10.1016/S0002-9440(10)65675-1 9777962 PMC1853044

[20] Golovina VA, Blaustein MP. Preparation of primary cultured mesenteric artery smooth muscle cells for fluorescent imaging and physiological studies. Nat Protoc. 2006;1(6):2681–7. Epub 2007/04/05. 10.1038/nprot.2006.425 . 17406524

[21] Li C, Hu Y, Mayr M, Xu Q. Cyclic strain stress-induced mitogen-activated protein kinase (MAPK) phosphatase 1 expression in vascular smooth muscle cells is regulated by Ras/Rac-MAPK pathways. The Journal of biological chemistry. 1999;274(36):25273–80. Epub 1999/08/28. . 10464250 10.1074/jbc.274.36.25273

[22] Banes AJ, Gilbert J, Taylor D, Monbureau O. A new vacuum-operated stress-providing instrument that applies static or variable duration cyclic tension or compression to cells in vitro. J Cell Sci. 1985;75:35–42. . 3900107 10.1242/jcs.75.1.35

[23] Bhatwadekar AD, Ghole VS. Rapid method for the preparation of an AGE-BSA standard calibrator using thermal glycation. Journal of clinical laboratory analysis. 2005;19(1):11–5. Epub 2005/01/13. 10.1002/jcla.20048 . 15645463 PMC6807708

[24] Simon R, Aparicio R, Housden BE, Bray S, Busturia A. Drosophila p53 controls Notch expression and balances apoptosis and proliferation. Apoptosis: an international journal on programmed cell death. 2014;19(10):1430–43. Epub 2014/05/27. 10.1007/s10495-014-1000-5 . 24858703

[25] Cai Y, Knight WE, Guo S, Li JD, Knight PA, Yan C. Vinpocetine suppresses pathological vascular remodeling by inhibiting vascular smooth muscle cell proliferation and migration. The Journal of pharmacology and experimental therapeutics. 2012;343(2):479–88. Epub 2012/08/24. 10.1124/jpet.112.195446 ; PubMed Central PMCID: PMCPmc3477207. 22915768 PMC3477207

[26] Dromparis P, Paulin R, Stenson TH, Haromy A, Sutendra G, Michelakis ED. Attenuating endoplasmic reticulum stress as a novel therapeutic strategy in pulmonary hypertension. Circulation. 2013;127(1):115–25. Epub 2012/11/15. 10.1161/CIRCULATIONAHA.112.133413 CIRCULATIONAHA.112.133413 [pii]. . 23149668

[27] Kalra M, Miller VM. Early remodeling of saphenous vein grafts: proliferation, migration and apoptosis of adventitial and medial cells occur simultaneously with changes in graft diameter and blood flow. J Vasc Res. 2000;37(6):576–84. Epub 2001/01/09. doi: 54091 [pii] 54091. . 11146412 10.1159/000054091

[28] Salzberg SP, Filsoufi F, Anyanwu A, von Harbou K, Karlof E, Carpentier A, et al Increased neointimal formation after surgical vein grafting in a murine model of type 2 diabetes. Circulation. 2006;114(1 Suppl):I302–7. Epub 2006/07/06. doi: 114/1_suppl/I-302 [pii] 10.1161/CIRCULATIONAHA.105.001339 . 16820590

[29] Lorusso R, Pentiricci S, Raddino R, Scarabelli TM, Zambelli C, Villanacci V, et al Influence of type 2 diabetes on functional and structural properties of coronary artery bypass conduits. Diabetes. 2003;52(11):2814–20. Epub 2003/10/28. . 14578301 10.2337/diabetes.52.11.2814

[30] Reimers MS, Zeestraten EC, van Alphen TC, Dekker JW, Putter H, Saadatmand S, et al Combined analysis of biomarkers of proliferation and apoptosis in colon cancer: an immunohistochemistry-based study using tissue microarray. International journal of colorectal disease. 2014;29(9):1043–52. 10.1007/s00384-014-1930-y . 24950792

[31] Mollereau B, Perez-Garijo A, Bergmann A, Miura M, Gerlitz O, Ryoo HD, et al Compensatory proliferation and apoptosis-induced proliferation: a need for clarification. Cell Death Differ. 2013;20(1):181 Epub 2012/06/23. 10.1038/cdd.2012.82 cdd201282 [pii]. 22722336 PMC3524636

[32] Ryoo HD, Bergmann A. The role of apoptosis-induced proliferation for regeneration and cancer. Cold Spring Harbor perspectives in biology. 2012;4(8):a008797 Epub 2012/08/03. 10.1101/cshperspect.a008797 ; PubMed Central PMCID: PMCPmc3405855. 22855725 PMC3405855

[33] Xia Y, Shen S, Verma IM. NF-kappaB, an active player in human cancers. Cancer immunology research. 2014;2(9):823–30. 10.1158/2326-6066.CIR-14-0112 25187272 PMC4155602

[34] Kravtsova-Ivantsiv Y, Shomer I, Cohen-Kaplan V, Snijder B, Superti-Furga G, Gonen H, et al KPC1-mediated ubiquitination and proteasomal processing of NF-kappaB1 p105 to p50 restricts tumor growth. Cell. 2015;161(2):333–47. 10.1016/j.cell.2015.03.001 . 25860612

[35] Simard E, Sollradl T, Maltais JS, Boucher J, D'Orleans-Juste P, Grandbois M. Receptor for Advanced Glycation End-Products Signaling Interferes with the Vascular Smooth Muscle Cell Contractile Phenotype and Function. PLoS One. 2015;10(8):e0128881 10.1371/journal.pone.0128881 26248341 PMC4527751

[36] Tsumagari K, Abd Elmageed ZY, Sholl AB, Friedlander P, Abdraboh M, Xing M, et al Simultaneous suppression of the MAP kinase and NF-kappaB pathways provides a robust therapeutic potential for thyroid cancer. Cancer letters. 2015;368(1):46–53. 10.1016/j.canlet.2015.07.011 26208433 PMC4555189

[37] Peng X, Li HX, Shao HJ, Li GW, Sun J, Xi YH, et al Involvement of calcium-sensing receptors in hypoxia-induced vascular remodeling and pulmonary hypertension by promoting phenotypic modulation of small pulmonary arteries. Molecular and cellular biochemistry. 2014;396(1–2):87–98. Epub 2014/07/27. 10.1007/s11010-014-2145-9 . 25063217

[38] Qu MJ, Liu B, Wang HQ, Yan ZQ, Shen BR, Jiang ZL. Frequency-dependent phenotype modulation of vascular smooth muscle cells under cyclic mechanical strain. J Vasc Res. 2007;44(5):345–53. 10.1159/000102278 . 17713348

[39] Chang S, Song S, Lee J, Yoon J, Park J, Choi S, et al Phenotypic modulation of primary vascular smooth muscle cells by short-term culture on micropatterned substrate. PLoS One. 2014;9(2):e88089 10.1371/journal.pone.0088089 24505388 PMC3913720

