# Supplementary figures and images for: Simultaneous Increases in Proliferation and Apoptosis of Vascular Smooth Muscle Cells Accelerate Diabetic Mouse Venous Atherosclerosis
Source: PLoS One. 2015 Oct 21;10(10):e0141375. doi: 10.1371/journal.pone.0141375 (PMC4619075; doi:10.1371/journal.pone.0141375)

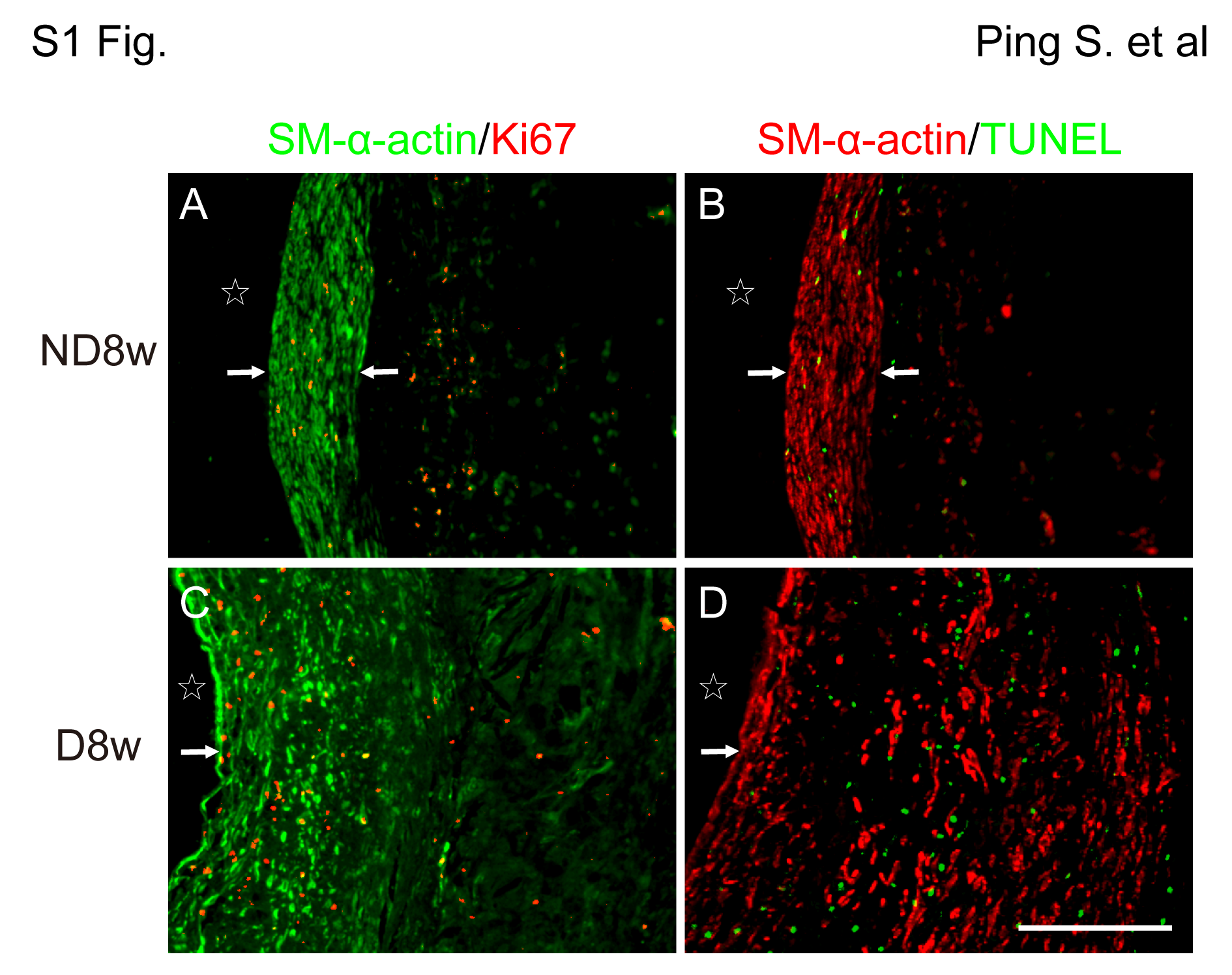

Supplement: S1 Fig — (A-D) Immunofluorescence detecting cell proliferation and apoptosis located mainly in SM-α-actin positive cells in both non-diabetic and diabetic mouse. Scale bars, 20 μm. (TIF) [file pone.0141375.s001.tif]

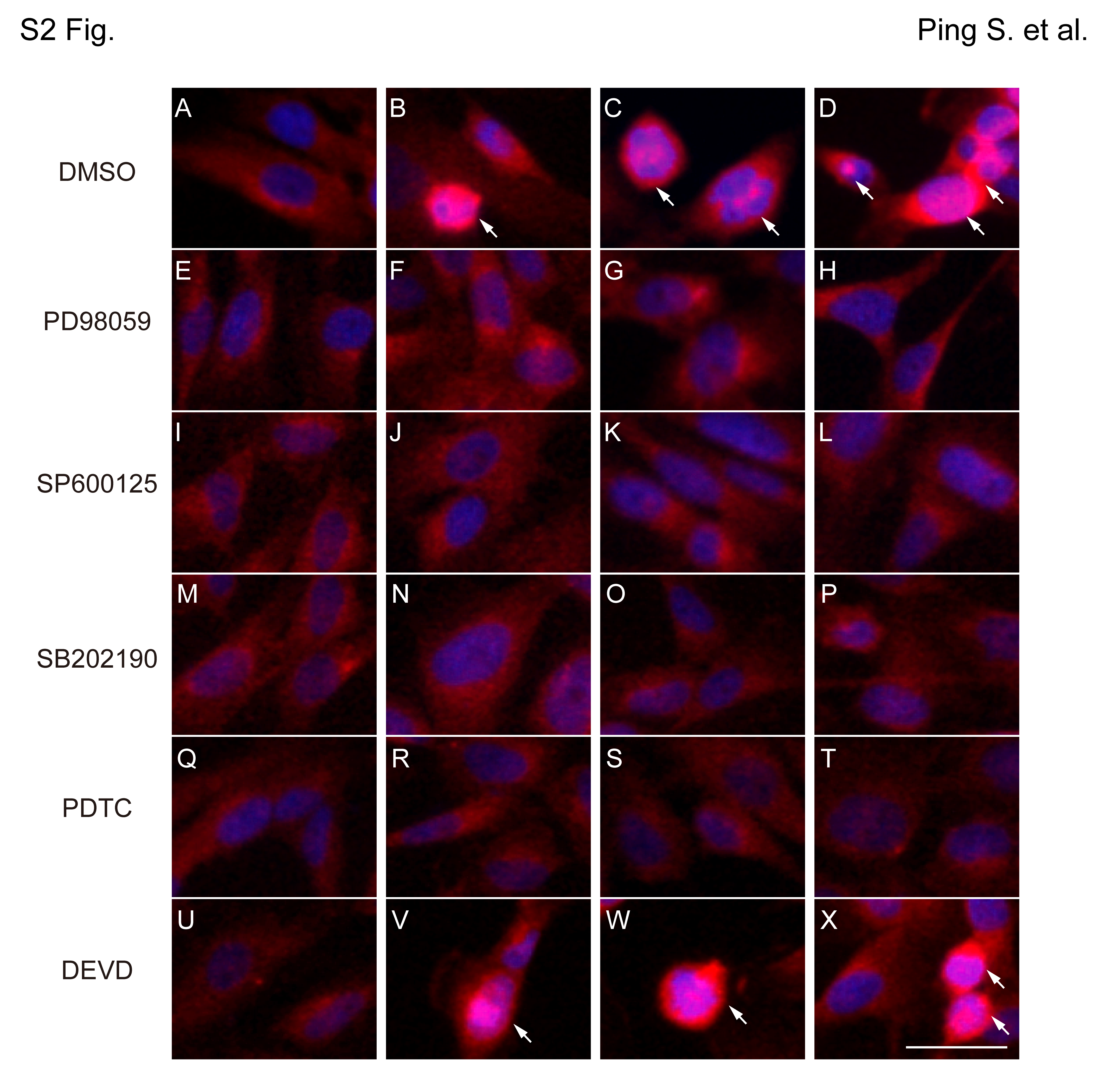

Supplement: S2 Fig — The cultured VSMCs were pretreated with DMSO, ERKs inhibitor PD98059, JNKs inhibitor SP600125, P38MAPK inhibitor SB202190, NF-κB inhibitor PDTC, and Caspase-3 inhibitor Z-DEVD-FMK for 1 h and then were treated with AGEs and/or for 30min. (A, B, C, D) Immunofluorescence showed either AGEs or SS could increase the NF-κB translocation (red, arrows), and the combination had a synergistic effect. (E-T) Immunofluorescence showed PD98059, SP600125, SB202190 and PDTC could significantly suppressed AGEs and SS induced increases of translocaiton. (U, V, W, X) Immunofluorescence showed Z-DEVD-FMK had no effect on AGEs and SS induced NF-κB translocation. Scale bars, 20 μm. (TIF) [file pone.0141375.s002.tif]

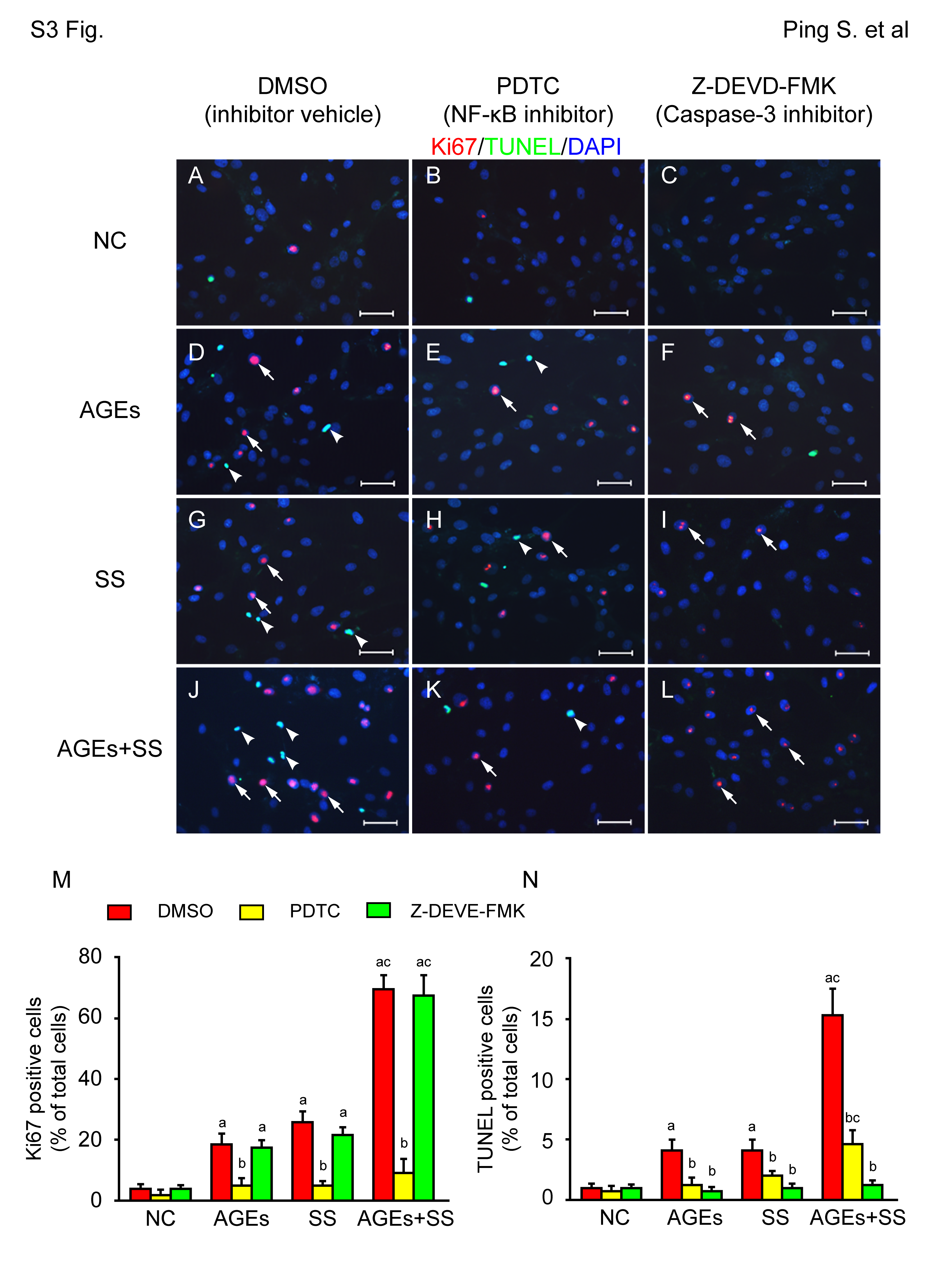

Supplement: S3 Fig — The cultured VSMCs were pretreated with DMSO, NF-κB inhibitor PDTC, and Caspase-3 inhibitor Z-DEVD-FMK for 1 h and then were treated with AGEs and/or for 1 h and continually cultured for 23 h. (A, D, G, J) Immunofluorescence showed either AGEs or SS could increase the cell proliferation (red, arrows) and apoptosis (green, arrowheads), and the combination had a synergistic effect. (B, E, H, K) Immunofluorescence showed PDTC significantly suppressed AGEs and SS induced increases of cell proliferation and apoptosis. (C, F, I, L) Immunofluorescence showed Z-DEVD-FMK significantly inhibited apoptosis of VSMCs while had no effect on cell proliferation. Scale bars, 20 μm. (M, N) Graph bars showed Ki67 and TUNEL positive ratios. All the experiments were independently repeated three times and shown as mean±SEM. a above bars are representing the p<0.05 compared to NC group, b represent the p<0.05 compared to DMSO group and c represent the p<0.05 compared to AGEs and SS group, n = 3 (a, b and c for p value<0.05). (TIF) [file pone.0141375.s003.tif]

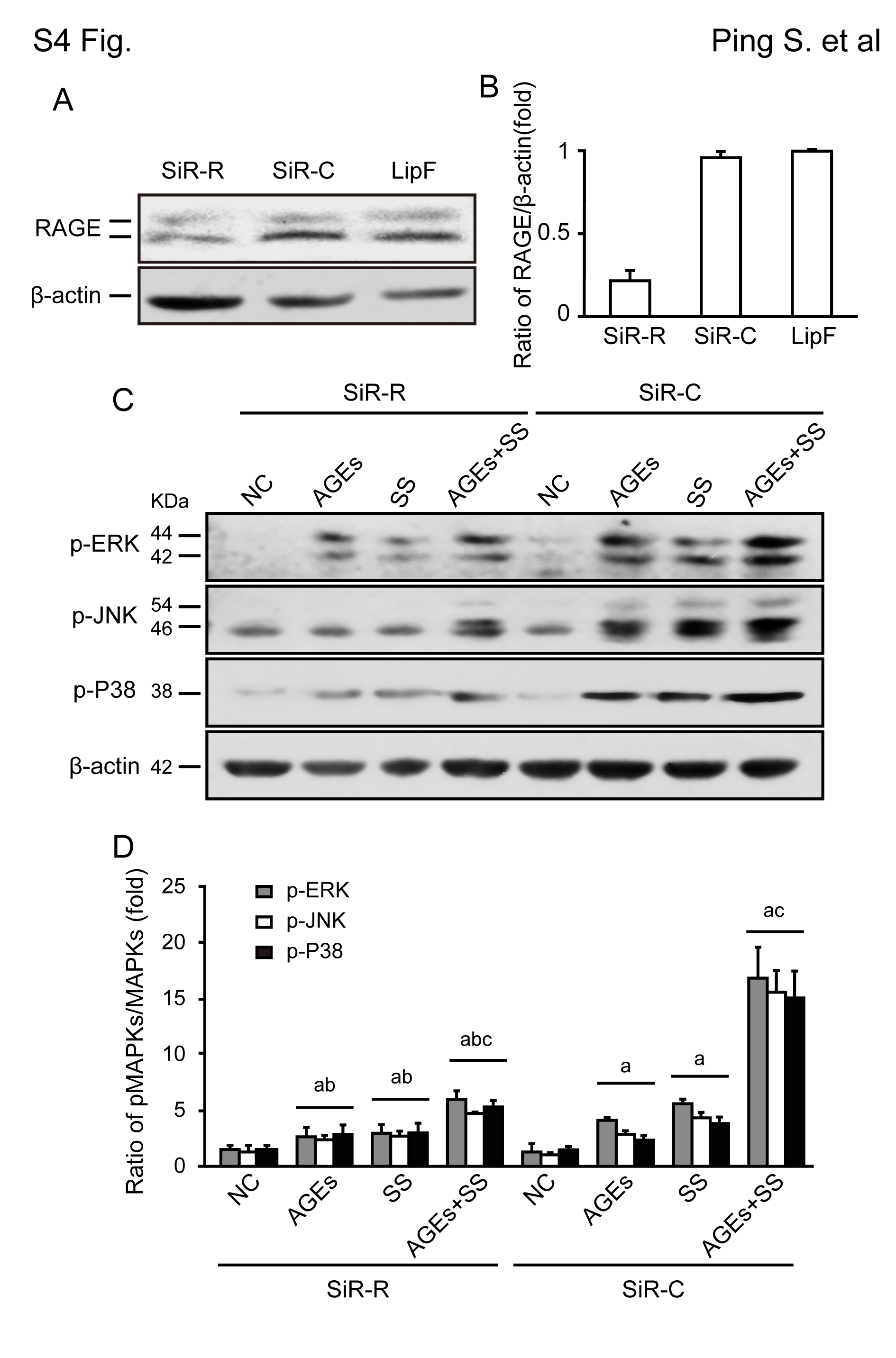

Supplement: S4 Fig — (A) Western blot analysis for RAGE detection of siRNA-RAGE transfected VSMCs. (B) Densitometry analysis of RAGE levels normalized with β-actin. (C) Western blot analysis for activation of MAPKs in siRNA-RAGE transfected VSMCs. (D) Densitometry analysis of MAPKs activation normalized with total MAPKs. All the experiments were independently repeated three times and shown as mean±SEM. a above bars are representing the p<0.05 compared to NC group, b represent the p<0.05 compared to siRNA-RAGE transfected group and c represent the p<0.05 compared to AGEs and SS group, n = 3 (a, b and c for p value<0.05). (TIF) [file pone.0141375.s004.tif]
